# Design on Power Factor Correction of a Digital Soft Switching Single-Phase Arc Welding Power Source

**DOI:** 10.3390/ma18092138

**Published:** 2025-05-06

**Authors:** Xiaoqing Lv, Minhao Jiang

**Affiliations:** 1 School of Materials Science and Engineering, Tianjin University, Tianjin 300350, China; 2 Tianjin Key Laboratory of Advanced Joining Technology, Tianjin 300350, China

**Keywords:** power factor correction, digitization, single-phase arc welding power source, soft switch

## Abstract

A power factor correction circuit for a single-phase arc welding power source using digital soft switching technology is proposed. The overall hardware structure of the system, the topology principle of the selected soft switch boost circuit, and the software design approach are discussed. The power factor correction results of the soft switch are verified under two conditions: electronic load and TIG arc welding. By using the electrical signals of the resonating capacitor and switching tube, it is confirmed that the circuit successfully achieved zero current conduction and zero voltage turn off. Through testing the power factor and efficiency of electronic loads at different powers, it was confirmed that the power factor can reach 0.985 or above, and the overall efficiency has been improved. Through TIG arc welding experiments under different welding currents, the corrected electrical signals are analyzed to verify the effectiveness of power factor correction for single-phase arc welding power.

## 1. Introduction

The power factor is an important index to measure the efficiency of electrical equipment [1,2]. A low power factor indicates that the reactive power of the equipment is large, and the system efficiency is low. China’s Energy Efficiency Limit Values and Energy Efficiency Classes for Electric Welding Machines (GB 28736 [3]), released in 2019, clearly stipulate the efficiency and power factor requirements for commercial electric welding machines. Due to the existence of nonlinear components and nonlinear arc loads in the main circuit, the arc welding power supply has serious current distortion and a low power factor, which not only causes interference to the power grid, but also reduces the efficiency of the welding machine. The power factor of an arc welding power source without power factor correction is usually around 0.7, whereas with PFC technology, the power factor can be increased to more than 0.98, significantly reducing grid losses and standby capacity requirements. Therefore, power factor correction of the arc welding power supply is particularly important [4,5]. At present, power factor correction of the arc welding power supply has been studied extensively, but this has been mostly limited to the use of the integrated analog chip hard switching method [6,7]. For example, Liu [8] studied power factor correction technology applied to an inverter welding machine based on the analog chip UC3854. In the boost part of the introduction of PFC technology, with a switching frequency of 100 kHz, the control circuit is based on the UC3854 peripheral circuit with average current control to achieve the switching off of the tube conduction. After PFC boosting, the voltage across the capacitor bank is sampled by a voltage divider resistor, input through the chip pins, and compared with the internal reference voltage of the chip. It is then output by the voltage error amplifier inside the chip. The synchronous signal input chip for rectified voltage detection and the output voltage signal of the voltage error amplifier are jointly applied to the input terminal of the internal multiplier. The output signal of the multiplier serves as the reference signal for current feedback control. After being compared with the detected value of the current, it is applied to the PWM modulator and driver through the current error amplifier to control the conduction and turn off of the switch tube, so that the low-frequency component of the input current is basically consistent with the waveform of the rectified voltage, greatly reducing current harmonics and improving the power factor of the circuit. As verified by the test, the introduction of PFC technology in the welder, the current waveform and voltage waveform for the same phase of the sinusoidal waveform, the total distortion rate of the current is greatly reduced, and the waveform has been improved to reduce the welder’s work on the pollution of the power grid. However, this approach has two shortcomings. First, the use of analog chips to achieve power factor correction has the advantages of fast response and high reliability, but there are problems such as low integration, poor programmability, and serious temperature drift; second, high switching loss, electromagnetic interference (EMI), and thermal management problems caused by hard switching are particularly prominent [9].

Therefore, we propose a power factor correction study of single-phase arc welding power supply based on digital soft switching technology. Digitalization brings the advantages of control flexibility and easy upgrade [10], and it is easy to integrate the control algorithm into the main control system of the arc welding power supply, thus reducing the cost [11,12]. The soft switching reduces the switching loss and improves the efficiency of the power factor correction circuit.

## 2. Overall Hardware Structure of the System

**Figure 1:**
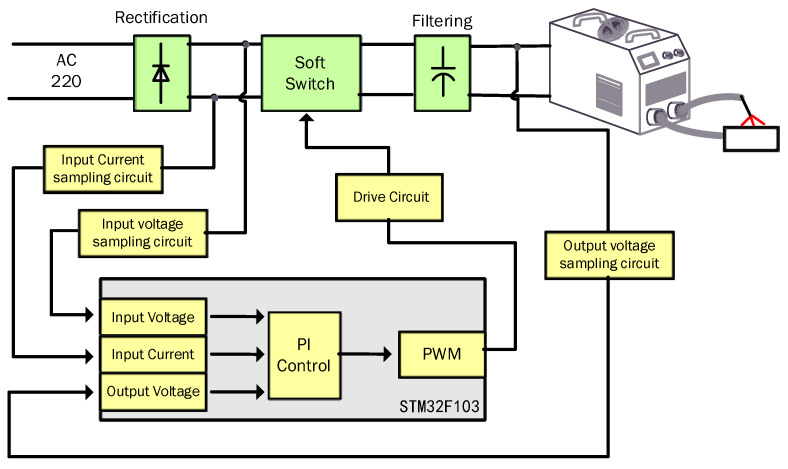
Overall diagram of digital soft switch welding machine.

The overall design of the digital soft switching power factor correction is shown in Figure 1, which includes three main parts: rectifier circuit, acquisition control circuit and soft switching circuit.

The rectifier circuit adopts a bridge rectifier to convert the AC mains power into DC power. The acquisition control circuit includes a 32-bit ARM microcontroller STM32F103ZET6 (STMicroelectronics, Geneva, Switzerland), isolated sampling circuits for input current, input voltage and output voltage, and a switch driver circuit. The Hall current sensor combined with differential circuitry is used to realize accurate current collection; the isolated operational amplifier combined with differential circuitry is used to realize input voltage and output voltage collection; the switching tube driver adopts gate isolation, which can effectively remove noise interference. The electrical signal data are collected to the microcontroller, and the PWM signal is output after PI operation, which controls the conduction and shutdown of the switching tube to realize the power factor correction.

The soft switch uses Boost as the main circuit topology and LC resonance to realize zero-current conduction and zero-voltage turn-off of the switch. The switching frequency is 100 kHz and the Boost target is 350 V.

## 3. Soft Switch Topology and Operating Principle

Zero switching PWM and zero switching PWM circuits are active soft switching technologies that reduce switching losses by adding auxiliary switches. However, the introduction of auxiliary switches not only increases the complexity of the main circuit and control circuit, and reduces the reliability of the system, but also increases costs. In addition, although the addition of auxiliary switching tubes reduces the loss of the main switching tube, the auxiliary switching tubes also bring new losses, which cannot be ignored. In addition, there is an RCD absorption circuit that can achieve a soft turn off effect. This is due to the presence of absorption capacitors, which form a charging process at the moment of turn off, delaying voltage recovery and reducing dv/dt, thus achieving soft turn off. Although the structure of the RCD absorption circuit is the simplest, the introduction of resistors to consume electrical energy leads to energy waste, thereby reducing the overall efficiency of the system. Especially in high-power applications, this loss becomes particularly pronounced. Therefore, RCD circuits often have the worst performance among various soft switching technologies and are not considered for use in this design. In contrast, passive lossless circuits do not rely on active or energy consuming components, but instead achieve energy transfer and feedback by adding passive components to the converter. This approach not only reduces energy loss, but also keeps the circuit structure relatively simple. Therefore, this article chooses to use passive lossless soft switching technology.

**Figure 2:**
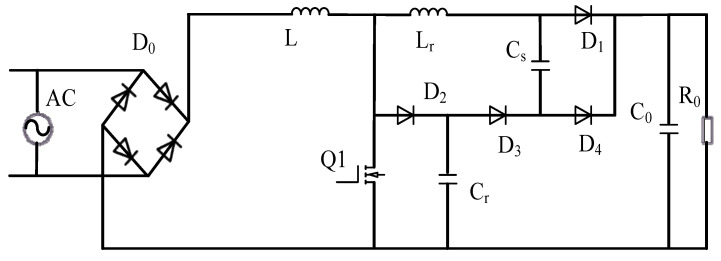
Boost passive lossless soft switching topology.

Figure 2 gives a schematic diagram of the soft switching Boost topology; in order to facilitate understanding, the rectifier circuit is also included. It consists of rectifier the bridge D_0_, inductor L, switch Q_1_, diodes D_1_, D_2_, D_3_, D_4_, resonant capacitor Cr, buffer capacitor Cs, and filter capacitor C_0_. The inductor Lr provides the zero-current conduction condition of the main switch Q_1_, and limits the reverse recovery current of the diode D_1_. The capacitor Cr provides the zero-voltage turn-off condition of switch Q_1_ [13,14,15].

**Figure 3:**
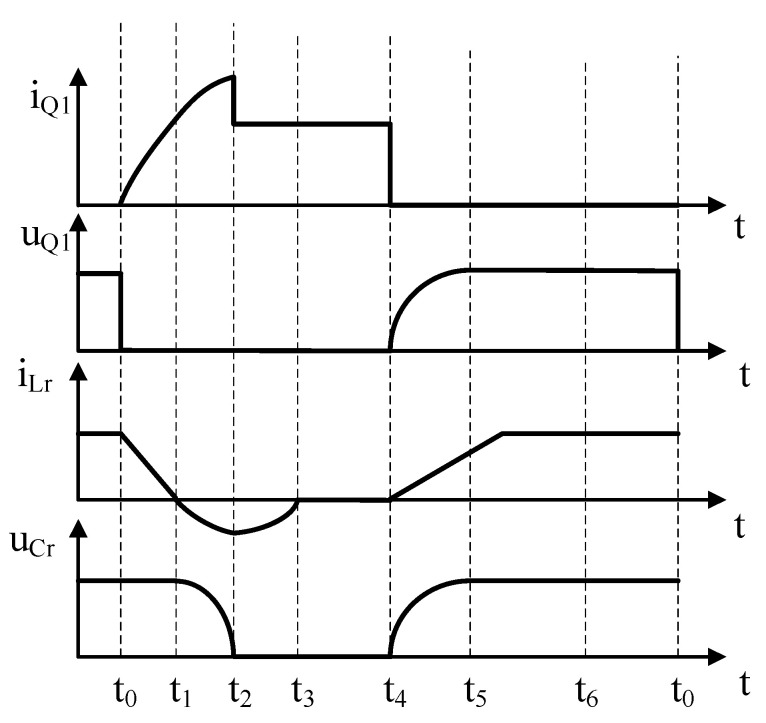
Various working states in soft switching circuits.

The operating states of the circuit are divided into seven stages for detailed analysis and the main operating states of the circuit are shown in Figure 3. For the sake of convenience, the voltage drop of the diode is ignored. In the figure, *i*_Q1_ is the current through the switch Q_1_, *u*_Q1_ is the voltage on both sides of the switch Q_1_, *i*_Lr_ is the current through the resonant inductor Lr, *u*_Cr_ is the voltage on both sides of the resonant capacitor Cr, *i*_L_ is the current through the inductor L, and *u*_0_ is the output voltage.

(1) Operating state 1: t < t_0_

This stage of the active switching tube Q_1_ is in the off state. The voltage across the resonant capacitor Cr *u*_Cr_ = *u*_0_, the voltage across the storage capacitor *u*_Cs_ = 0, and the current across the resonant inductor Lr is *i*_Lr_ = *i*_L_.

(2) Operating state 2: t_0_ ≤ t < t_1_

At t_0_, the switch Q_1_ turns on, and since the current through the inductor Lr cannot be changed abruptly, the current i_Q1_ through the switch starts to rise from zero, thus realizing zero-current conduction of the switch. At this time, the current through the inductor *i*_Lr_ decreases linearly. Since the current *i*_Q1_ = *i*_L_ − *i*_Lr_, through the switch, the current i_Q1_ rises at the same rate as the current i_Lr_ falls. At the same time, Lr suppresses the reverse recovery current of the diode D_1_ and attenuates the voltage spike during reverse recovery. At t_1_, current *i*_Lr_ drops to zero. The currents through the switch i_Q1_ and the resonant inductor Lr are:

(1) $$i_{Q1}=\frac{u_{0}}{L_{r}}\left(t-t_{0}\right)$$

(2) $$i_{Lr}=i_{L}-\frac{u_{0}}{L_{r}}\left(t-t_{0}\right)$$

(3) Operating state 3: t_1_ ≤ t < t_2_

At t_1_, after the current *i*_Lr_ drops to zero, the resonant capacitor Cr starts to resonate with Cs and Lr, and the resonant circuit contains Cs, D_3_, Cr, Q_1_ and Lr. The energy in Cr starts to be transferred to the buffer capacitance Cs and the inductor Lr, the voltage *u*_Cs_ in Cs rises from zero, and the current in Lr increases in the opposite direction from zero. At this time, from the initial voltage magnitude u0 of the resonant capacitor Cr, *i*_Lr_, *u*_Cr_ and *u*_Cs_ can be calculated.

(3) $$L_{r}\frac{di_{Lr}}{dt}+u_{Cs}-u_{Cr}=0$$

(4) $$C_{S}\frac{du_{Cs}}{dt}=i_{Lr}$$

(5) $$-C_{r}\frac{du_{Cr}}{dt}=i_{Lr}$$

(4) Operating state 4: t_2_ ≤ t < t_3_

At t_2_, *u*_Cr_ = 0, and the current *i*_Lr_ will approach its reverse maximum. At this point, the inductor Lr resonates with the buffer capacitor Cs, and the resonant circuit contains Lr, D_2_, D_3_, and Cs. The resonance transforms the magnetic energy in Lr into energy in Cs, and the voltage *u*_Cs_ across Cs continues to rise. The voltage *u*_Cs_ across Cs continues to rise until the energy in Lr is discharged. The expressions for *i*_Lr_ and *u*_Cs_ can be calculated as follows:

(6) $$L_{r}\frac{di_{Lr}}{dt}+u_{Cs}=0$$

(7) $$\;C_{s}\frac{du_{Cs}}{dt}=i_{Lr}$$

(5) Operating state 5: t_3_ ≤ t < t_4_

In this stage, the soft switching circuit is not involved in the circuit operation. The energy of the resonant capacitor Cr has been transferred to the buffer capacitor Cs, *u*_Cr_ = 0, and the voltage on both sides of Cs remains at its maximum value. The main circuit operates in normal boost mode.

(6) Operating state 6: t_4_ ≤ t < t_5_

At t_4_, the switch Q_1_ is turned off, and since the voltage *u*_Cr_ = 0 on both sides of the resonant capacitor Cr, the switching voltage u_Q1_ is clamped to zero as the capacitor voltage cannot be changed abruptly. Subsequently, u_Q1_ will rise together with *u*_Cr_, thus realizing zero-voltage turn-off of the switch. At the same time, the energy in the buffer capacitor Cs is fed back to the load. The feedback loop consists of the power supply, L, Lr, Cs, D_4_, and the load.

(7) Operating state 7: t_5_ ≤ t < t_6_

In this stage, the energy in the buffer capacitor Cs continues to be fed back to the load. At t_6_, *u*_Cs_ = 0, the feedback is complete. Then the diode D_1_ begins to conduct, back to the initial operating state, and the main circuit enters the normal Boost mode of operation.

(8) $$i_{L}=i_{Lr}\left(t\right)+i_{Cr}\left(t\right)$$

(9) $$u_{Cr}\left(t\right)=\mathrm{Lr}\frac{di_{Lr}\left(t\right)}{dt}-u_{Cs}\left(t\right)$$

(10) $$i_{Cr}\left(t\right)=Cr\frac{du_{Cr}\left(t\right)}{dt}$$

(11) $$i_{Lr}\left(t\right)=-Cs\frac{du_{Cs}\left(t\right)}{dt}\;$$

From the above analysis process, it can be seen that [13], the soft switching circuit realizes the zero-current conduction and zero-voltage turn-off of the switching tube, which reduces the switching loss of the switching tube, improves the efficiency of the converter, and increases the service life.

Considering the soft switching realization conditions, according to references [16,17], for the design of the soft switching parameters, although a larger Cr is beneficial to reduce the losses of the switching tube, the value of Cr should not be too large, which will cause the effective duty cycle to decrease due to the long commutation time, thus affecting the power factor correction effect, and also increase the transient current of the switching tube during the on-time.

Based on the above circuit topology and structure analysis, the rectifier bridge D_0_ is selected as GBJ5010, the switching tube Q_1_ adopts the silicon carbide field effect tube HC3M0015065D, the diodes D_1_, D_3_~D_4_ adopt the silicon carbide diode HC3D30065H, and the D_2_ adopts IDH10G65C6, the inductor of the main circuit is L = 0.4 mL, the resonance capacitance Cr = 4.7 nF, the buffer capacitance Cs = 4.7 nF, the inductance Lr = 1 μH, and the output filter capacitance C_0_ = 1 mF.

## 4. Software Design

**Figure 4:**
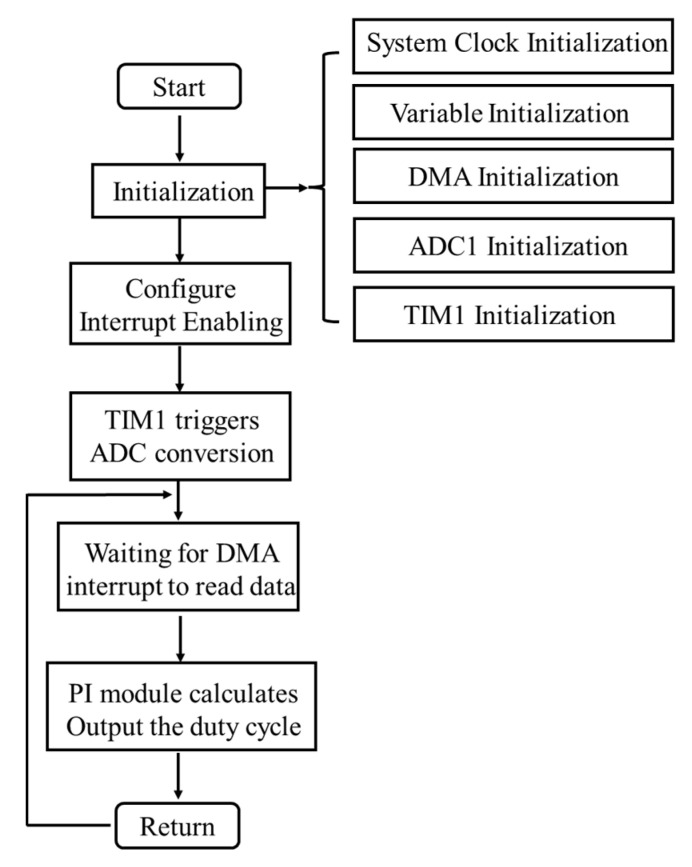
Main program of the system.

Software control is the key to power factor correction in digital welding machines. It mainly realizes the current control in the inner loop and the voltage control in the outer loop. The purpose of the former is to make the input current follow the sinusoidal input voltage, while that of the latter is to keep the output filtered voltage constant. The main program flow is shown in Figure 4. After the program starts, it first enters the initialization settings, including the initialization of the system clock, variables, ADC, DMA, and timer TIM1. The program starts with initialization settings, including the system clock, variables, ADC, DMA and timer TIM1 initialization. Then the timer TIM1 is started, so that its PWM triggers the ADC. Next, the acquisition is completed and stored in the DMA buffer, and the DMA interrupt has been read. After obtaining the input and output signal data, the double PI module operation (inner loop current, outer loop voltage) is carried out, followed by updating the PWM duty cycle, and then continuing to wait for the DMA interrupt readings, and so on.

The process continues as follows: Configure pins 1, 4 and 5 of GPIOA as the signal acquisition ports for input voltage, input current and output voltage, respectively. Set the ADC clock for the APB2 (high-speed peripheral bus) 6 divisions, that is, 12 MHz. AD the entire conversion time by the sampling time and analog-to-digital conversion time, in which the sampling time is set to the fastest, 1.5 clock cycles and the analog-to-digital conversion time of 12.5 clock cycles. Therefore, it takes 14 clock cycles to convert one piece of data, the time is about 1.16 μs, and the total time for sampling once for 3 channels is about 3.48 μs. After each AD conversion, the data are stored in the DMA buffer to be read by interrupt mode. Since the switching frequency is 100 kHz and the cycle time is 10 μs, the data are collected five times every two cycles. By averaging the data over the five cycles, the electrical signals are accurately acquired within the cycle. At the same time, an acquisition completion mark is given to indicate that the PI operation can be performed, and the acquisition is stopped to wait for the next trigger. In order to minimize the acquisition error and ensure that the initial moment of each acquisition is the same, the AD converter is designed to start triggering by the rising edge of the pulse width.

Since the power factor correction has an inner-loop current control and an outer-loop voltage control, a double closed-loop PI control is required [18,19,20]. The control error of the inner loop is obtained by comparing the input voltage with the product of the input current and a fixed coefficient, while the control error of the outer loop is obtained by comparing the target voltage with the measured output voltage. Since the specific object of dual-loop control is the duty cycle of the switching tubes, if the dual feedbacks are controlled at the same frequency, they will affect each other. Considering the power factor correction as the most important goal of this study, the control frequency is much higher than that of the voltage loop. Since the acquisition takes two cycles, the current loop control frequency is 50 kHz half of the main circuit inverter frequency. The duty cycle of the PWM is changed every two cycles. The voltage loop is adjusted once every 800 current loops. The control frequency is 62.5 Hz. Through debugging, the current loop coefficients are P = 0.2 and I = 0.12, while the voltage loop coefficients are P = 0.04 and I = 0.3.

## 5. Results

Following the design of the hardware and software, the power factor correction circuit is verified on Chroma’s 63200E electronic load (Chroma ATE Inc., Suzhou, China) and Wowei Electric’s single-phase TIG arc welder, the WS-250S (Woweld Electric, Shenzhen, China).

**Figure 5:**
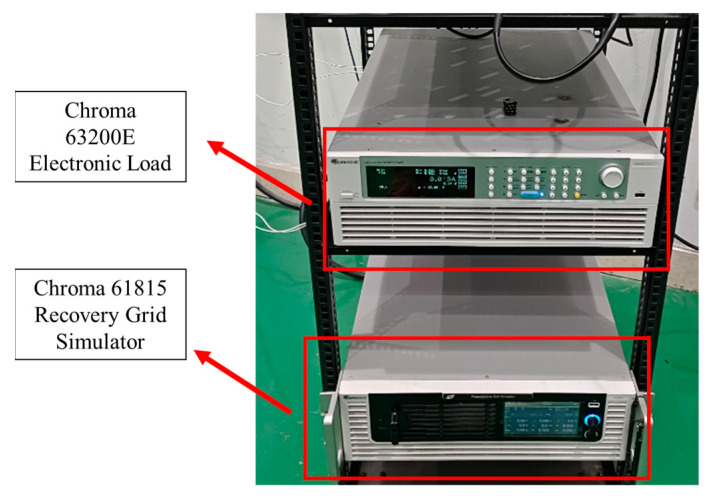
Simulation test platform.

The electronic load test platform is shown in Figure 5, which consists of an electronic load and an analogue power supply. The electronic load is operated using Chroma’s 63200E-210-600 model (Chroma ATE Inc., Suzhou, China), while the analogue power supply utilizes Chroma’s three-phase energy recovery 61815 model (Chroma ATE Inc, Suzhou, China), which employs a single-phase form.

**Figure 6:**
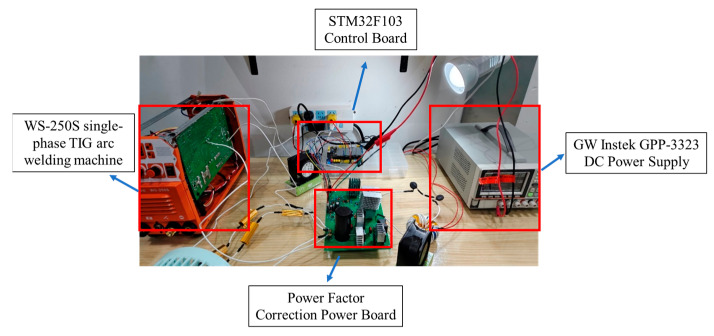
Welding test platform.

The welding test platform, as illustrated in Figure 6, comprises the following components: The welding machine utilizes a Woweld Electric single-phase TIG arc welding machine model WS-250S, accompanied by a power factor correction board designed to substitute for the input rectifier component of the welding machine. It has two working modes: manual welding and argon arc welding. Its rated input capacity is 5.6 kVA. The output current range of TIG welding is 10 A–195 A. The rated working voltage is 20 V. The welding machine efficiency can reach 85%. During welding, 99.999% pure argon gas is used as the protective gas, and the plate is a 1 cm thick steel plate. The power supply for the power factor correction board is provided by a GPP-4323 linear power supply. The power factor is measured using a Victory Instruments Clamp Power Meter VC 7300M (Victor Instrument, Shenzhen, China).

### 5.1. Soft Switch

**Figure 7:**
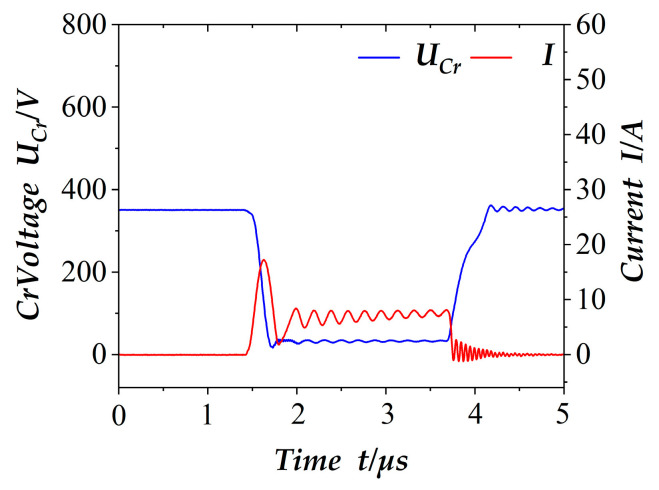
Resonant capacitance and switching transistor status.

In the electronic load operation mode, the power of the circuit board is 2000 W, through the resonant circuit components and the voltage and current signals across the switching tube to verify the effectiveness of soft switching. The resonant capacitor Cr voltage and switch current changes are shown in Figure 7. It can be seen that, when the switch is on, the resonant capacitor begins to release energy and the current flow through the switch rises until the peak current; the resonant capacitor Cr in the energy is also discharged, which coincides with the above in the operating state 3. At this point, the resonant circuit consists of Cs, D_3_, Cr, Q_1_, and Lr. After that, the resonant capacitor Cr does not participate in the resonance of the inductor Lr and the buffer capacitor Cs. It should be noted that there are oscillations in the switching current due to the existence of resonant inductance, parasitic capacitance, and filter capacitance, and the voltage on the buffer capacitor Cs is not fixed, so in the theoretical analysis of the on-state or off-steady state process, the experimental waveforms show several oscillations and then stabilize; the existence of this phenomenon is normal [21], and it will not affect the effect of switching tube on-off and soft switching.

The 50 Hz alternating current (AC) is rectified to 100 Hz pulsating direct current (DC), resulting in a switching tube that completes one cycle within 10 ms. The duty cycle in one cycle of power factor correction is subject to real-time change. Generally, the larger the input voltage, the smaller the duty cycle [22]. In order to comprehensively examine the entire cycle of soft switching, 2 ms, 5 ms, 7 ms three moments is selected. It is evident that the smallest duty cycle is 5 ms and the largest is 2 ms.

**Figure 8:**
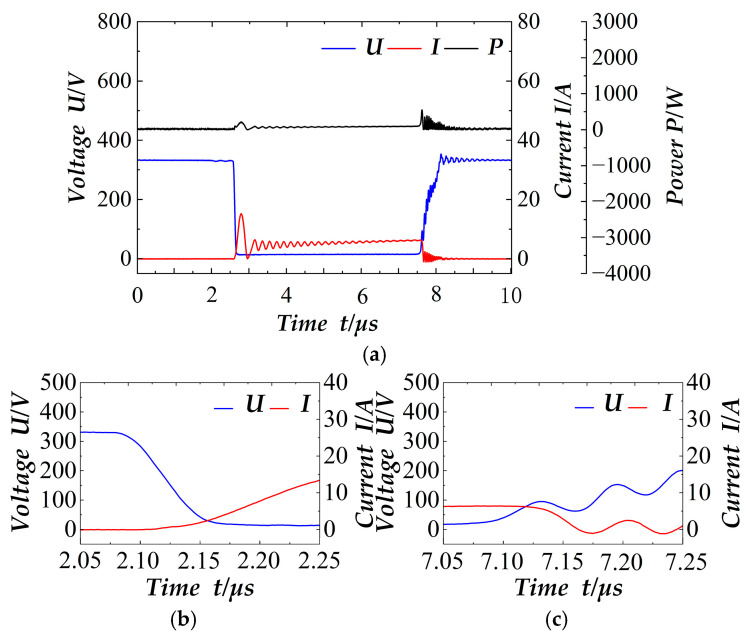
2 ms soft switch signal: (**a**) Soft switch electrical signal; (**b**) Details of the switching-on area; (**c**) Details of the switching-off area.

The electrical signals across the switch terminals at 2 ms in the cycle are shown in Figure 8. These are the single switching tube electrical signals, the local details of conduction, the local details of switching off, and the conduction time is about 5 µs. In this case, the power is taken as the absolute value of the product of the voltage and current. It is evident that the current rises gradually and the voltage is at zero value during the on-time, thus demonstrating the effect of zero-current conduction. During off-time, the resonant capacitor Cr hinders the voltage change, resulting in the intersection with the current being at the smaller value of the voltage, thereby achieving the effect of zero-voltage turn-off. In conclusion, the soft switching effect of the design is achieved. Furthermore, Figure 7 and Figure 8 demonstrate that the voltage drop during the switching period is 350 V, which is precisely the target voltage designed by the outer loop control, thereby indicating that the outer loop control has achieved the target with great precision.

**Figure 9:**
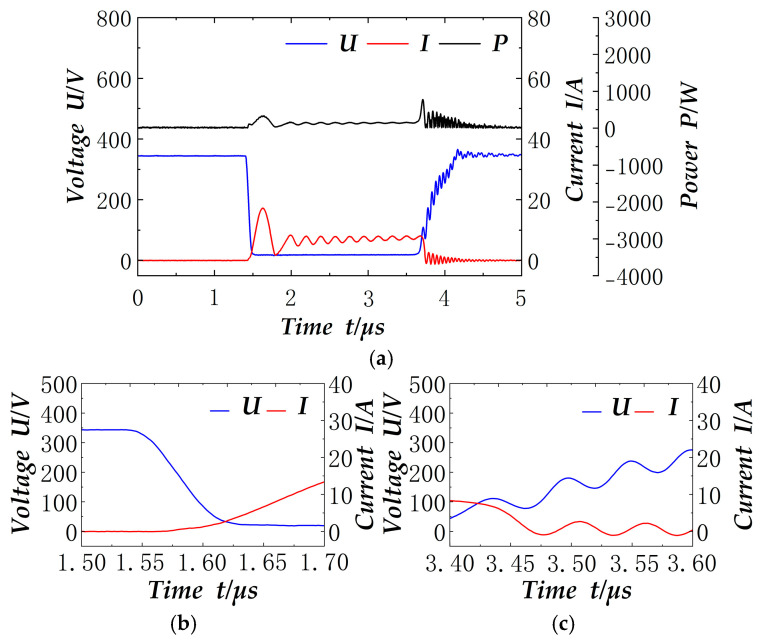
5 ms soft switch signal: (**a**) Soft switch electrical signal; (**b**) Details of the switching-on area; (**c**) Details of the switching-off area.

At the 5 ms switching tube signals shown in Figure 9, the on-time is about 2.3 µs. As the current rises slowly in the zero value, the zero-current conduction effect is obvious; shutdown and the resonant capacitor Cr impede the voltage change, so that the intersection with the current is also in the voltage of the smaller value, to achieve the effect of zero-voltage shutdown. The figure also shows the switching tube shutdown moment of the voltage of 350 V, similar to the above, indicating that the outer loop voltage control has reached the target.

**Figure 10:**
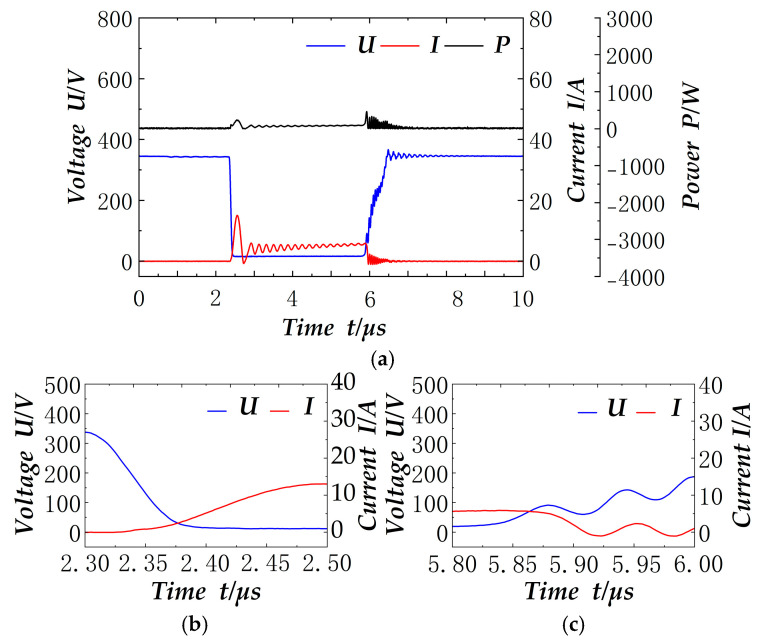
7 ms soft switch signal: (**a**) Soft switch electrical signal; (**b**) Details of the switching-on area; (**c**) Details of the switching-off area.

The signals at 7 ms across the switching tube are shown in Figure 10, The on-time of this signal is approximately 3.6 ms. The same on-time current rises slowly in the zero value, thereby demonstrating the zero-current conduction effect. Shutdown is observed, and the resonant capacitance Cr impedes the voltage change, resulting in an intersection with the current that is also in the voltage being small. This is achieved in order to achieve the effect of zero-voltage shutdown. Similarly, it can be seen from the figure that the outer loop control target reaches 350 V.

**Figure 11:**
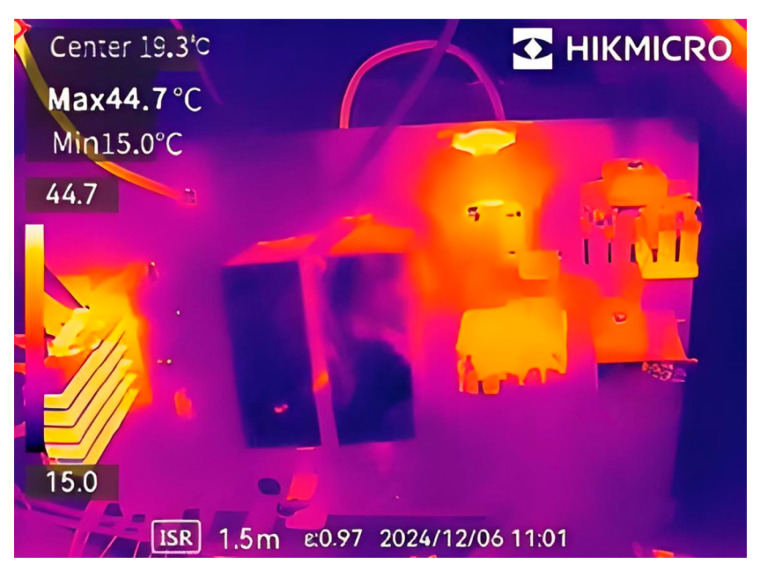
Thermal imaging analysis of power board.

Thermal imaging analysis on the power board under full load condition for a period of time was performed using a thermal imaging instrument, as shown in Figure 11. The highest temperature between the boards is 44.7 °C, mainly concentrated in the diode components of the rectifier bridge, main circuit, and resonant circuit, which are high-temperature areas, but the temperature is still within a controllable and safe range. However, due to the use of soft switch design and heat sink, the temperature at the switch tube is relatively low.

### 5.2. Electronic Load

**Figure 12:**
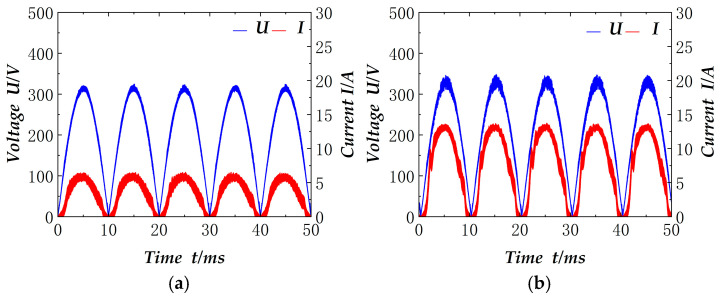
Electronic load power factor correction electrical signal: (**a**) 150 Ω; (**b**) 60 Ω.

The electronic load is set in the mode of constant resistance, and the resistance is varied to verify the effect of factor correction at different powers. To facilitate the actual collection, the collected electrical signals are after the rectifier bridge. Figure 12 shows the current and voltage signals measured after the rectifier bridge when the electronic load resistance is set to 150 Ω and 60 Ω respectively, and the power is about 817 W and 2041 W. As illustrated in the figure, the rectified currents and voltages exhibit congruent waveforms and are situated in the same phase. Furthermore, the power factor has undergone correction, achieving a value close to 0.985.

**Figure 13:**
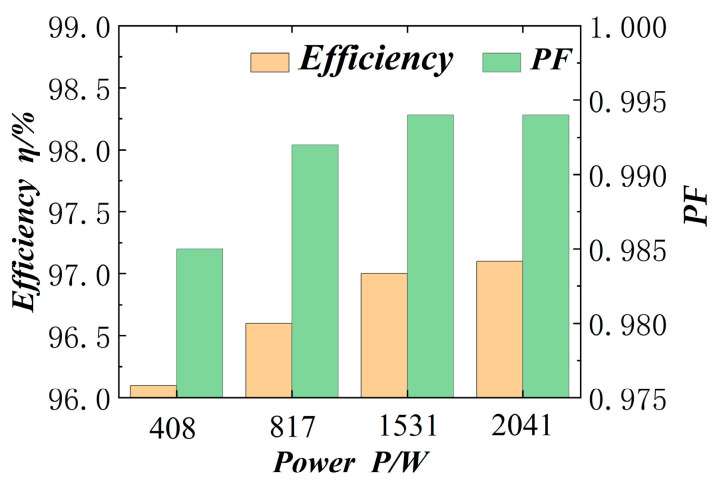
Circuit efficiency and power factor.

We adjust the setting resistance of the electronic load, so that the whole circuit ranges from 500 W to 2000 W, and then tested and compared the input and output power, to obtain the efficiency of the circuit and the power factor at each power, as shown in Figure 13. The efficiency of the circuit is maintained at 96–97%, as can be seen from the figure. Compared with the conventional hard-switched Boost circuits [23], which have an efficiency of 94.7–95.3% at 500 W to 2000 W, the overall efficiency of the circuit is improved, and the power factor is maintained above 0.985.

### 5.3. Welding Test

**Figure 14:**
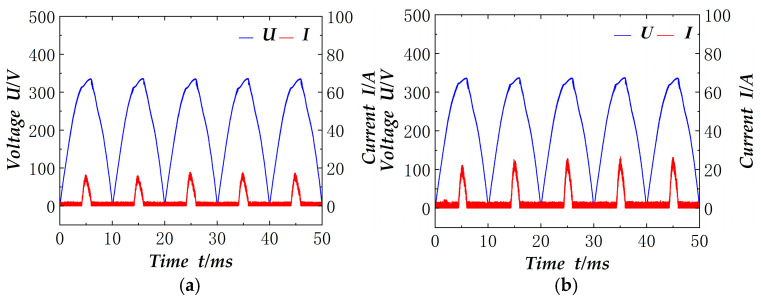

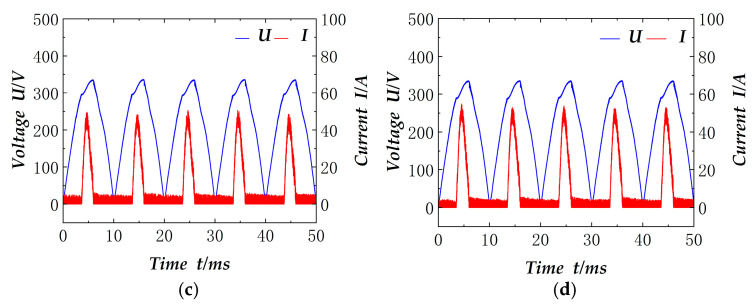
Input electrical signal of non-power factor correction welding machine: (**a**) 30 A; (**b**) 50 A; (**c**) 90 A; (**d**) 110 A.

Similar to the electronic load, the input signals are taken after the rectifier bridge in the welding test. Firstly, the welding machine without the power factor correction circuit is shown in Figure 14. The figure shows the welding current of 30 A, 50 A, 90 A, 110 A, and the current and voltage signals after the rectifier bridge. It is evident that the current is not continuous, the distortion is serious, and the actual power factor measured is 0.3–0.5. It should be noted that the voltage in the figure is not strictly a sinusoidal half-wave, which is related to the load state of the grid at that time.

**Figure 15:**
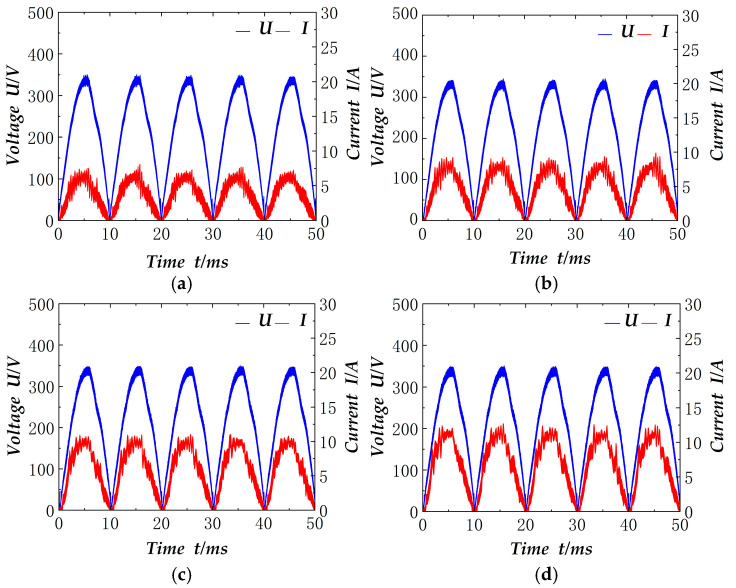
Input electrical signal of welding machine after power factor correction: (**a**) 30 A; (**b**) 50 A; (**c**) 90 A; (**d**) 110 A.

The power factor correction circuit is connected to the welder. It replaces the original rectifier part of the welder. As demonstrated in Figure 15, the incorporation of the power factor correction circuit results in alterations to the electrical signals received by the welder. The figure illustrates the voltage and current waveforms subsequent to the rectifier bridge for TIG welding currents of 30 A, 50 A, 90 A and 110 A, respectively. The corresponding actual output power is 1030 W, 1320 W, 1654 W and 1985 W, respectively. A comparison with the circuit lacking power factor correction (as shown in Figure 14) reveals the efficacy of the rectification process, with the current waveform exhibiting near-identical characteristics to the voltage waveform. The current remains undistorted, and the power factor in the measurement can attain a maximum of 0.99. Furthermore, it is evident from Figure 14 and Figure 15 that the voltage waveforms are not strictly sinusoidal half-waves, as evidenced by the presence of distortion, and the current waveforms in Figure 14 are also similarly distorted, which further verifies the effectiveness of the circuit’s power factor correction.

However, the current waveform in Figure 15 is marginally larger than that of the electronic load in Figure 12. This discrepancy can be attributed to the inherent instability of the arc combustion process during welding, leading to variations in the welding load. The welding load is known to be less stable than the electronic load, necessitating constant adjustments to the current control system. Consequently, the current fluctuation is observed to be slightly larger than that of the electronic load.

## 6. Conclusions

Based on the background of China’s current energy efficiency requirements for welding machines, a digital soft switching power factor correction circuit is designed for the characteristics of single-phase arc welding power supply. The hardware structure, device selection and software design are presented. The following conclusions were reached:

- The soft switching test was selected for three different moments within a single cycle, all of which were verified, and the results showed that the soft switching goal was achieved.
- The electronic load results show that, for the power from 500 W to 2000 W, the power factor correction can be more than 0.985, and the circuit efficiency can be more than 96%.
- For the TIG welding current of 30 A to 110 A, the power factor is up to 0.99, and the inner loop current control fluctuation is slightly larger than the electronic load.

## Data Availability

The original contributions presented in this study are included in the article. Further inquiries can be directed to the corresponding author.
